# Introduction of a novel optical measurement system for dynamic spinal curvature analysis

**DOI:** 10.1186/1748-7161-8-S2-O31

**Published:** 2013-09-18

**Authors:** Marcel Betsch, Walter Rappr

**Affiliations:** 1 Oregon Health & Science University, Portland, OR, USA

## Background

Rasterstereography is a method for stereophotogrammetric surface measurements of the back based on moiré topography[1]. In contrast to all other optical devices, rasterstereography allows an analysis not only of the back surface but also of the underlying spine. This is possible by the use of a spine model, which was created by Turner-Smith[2,3]. So far rasterstereography only allows static measurements. However, it would be of great value to be able to also evaluate the spine and its segments under dynamic conditions.

## Purpose

The purpose of this study was to evaluate the accuracy of the marker detection of a novel spine and surface topography system that allows measurements under dynamic conditions.

## Methods

A new rasterstereographic device (Diers, Germany) was evaluated, by comparison with the gold standard in motion analysis the VICON system. 12 flat infrared markers were adhered to a wooden plate and on the backs of 8 test subjects. By linking the markers, 4 triangles were defined, and the sides of each triangle were acquired under static and dynamic conditions while subjects walked on a treadmill.

## Results

On the wooden plate the sides of the 4 triangles were measured by hand with a measuring tape and by the two optical systems. Here, the rasterstereograph showed a higher accuracy in marker detection than the VICON system (0.13 ± 0.84mm vs. 0.73 ±0.71mm). Under dynamic conditions when the subjects walked on a treadmill, the rasterstereographically-measured sides of all four triangles were compared with the sides measured by the VICON system. No significant differences (p>0.05) were found in the marker detection between the systems, as the marker detection differed between 0.07 - 1.1% for all sides of the four triangles.

## Conclusions and discussion

The accuracy of the rasterstereograph is superior on a wooden plate and comparable during dynamic measurements to the VICON system, with the advantage that it calculates a three-dimensional surface map and also allows the analysis of the underlying spine. The measurements made under dynamic conditions can be factored into the spinal model to allow an analysis of the scoliotic spine during motion.
